# FAV-DenoiseNet: An Audio–Visual Speech Enhancement Framework Based on Conditional Flow Matching and Visual Encoding

**DOI:** 10.3390/s26134175

**Published:** 2026-07-02

**Authors:** Xuan Fu, Lulu Qin, Weijing Liu, Mingchen Sun, Dadong Wang

**Affiliations:** 1 School of Computer Science, Jilin Normal University, Siping 136000, China; 243110865@mails.jlnu.edu.cn (X.F.); qinlulu@mails.jlnu.edu.cn (L.Q.); lwjing@mails.jlnu.edu.cn (W.L.); 2 School of Computer Science and Technology, Jilin University, Changchun 130012, China; mcsun20@mails.jlu.edu.cn; 3 Jilin Provincial Key Laboratory for Numerical Simulation, Siping 136000, China

**Keywords:** audio–visual speech enhancement, conditional flow matching, discriminative prior denoising, multi-scale cross-modal attention, single-step inference

## Abstract

Audio–visual speech enhancement aims to recover clean speech by jointly using noisy acoustic signals and synchronized visual cues. Although diffusion-based methods achieve promising restoration performance, their multi-step sampling causes high inference latency and computational cost, limiting real-time deployment. To address this issue, this paper proposes FAV-DenoiseNet, a two-stage framework based on discriminative prior denoising and conditional residual flow matching. The first stage uses a pre-trained discriminative denoising network to suppress dominant noise and provide a structurally stable speech prior. The second stage reformulates enhancement as residual compensation between the first-stage output and the clean speech spectrum instead of directly predicting the entire clean spectrum. A conditional flow-matching network estimates the residual from zero-residual initialization through single-step inference, reducing generative sampling cost. Multi-scale cross-modal attention provides adaptive visual guidance for audio refinement at different resolutions. A residual-controlled fusion strategy preserves the stable structure recovered by the first stage while compensating for residual noise, high-frequency details, and weak speech components. The experimental results show that FAV-DenoiseNet achieves PESQ, ESTOI, and SI-SDR scores of 2.805, 0.775, and 12.480 dB on VoxCeleb2 and 3.157, 0.876, and 13.281 dB on GRID, respectively, with an RTF of 0.086. These results demonstrate that the proposed framework effectively balances enhancement quality, detail restoration, and real-time inference efficiency.

## 1. Introduction

Speech enhancement aims to recover clean and natural target speech from noisy signals, which is critical for applications such as speech communication, video conferencing, hearing aids, automatic speech recognition, and human–computer interaction. In real-world scenarios, speech signals are often corrupted by background noise, competing speakers, reverberation, and recording device variability, resulting in degraded quality and reduced intelligibility. Under low-SNR or non-stationary noise conditions, fine speech details may be severely masked, posing significant challenges for accurate restoration.

Traditional approaches rely on noise assumptions or prior SNR estimation. While effective in stationary environments, they often leave residual noise or introduce speech distortion under complex acoustic conditions. Deep learning methods, including convolutional, recurrent, convolutional-recurrent, and Transformer-based architectures, have advanced time–frequency mask estimation, spectral mapping, and complex spectral modeling. Complex spectral models, which account for both magnitude and phase, preserve speech structure more effectively than magnitude-only approaches. Nevertheless, audio-only methods remain limited: when noise is strong or the target speech is heavily masked, audio features alone cannot reliably recover weak speech components or high-frequency details, often leading to over-smoothing or over-suppression.

Visual cues provide complementary information, as lip movements and mouth shapes are tightly coupled with articulation and remain unaffected by acoustic noise. When audio information is unreliable, visual features can constrain speech reconstruction. Audio–visual speech enhancement (AVSE) leverages this property to improve quality and intelligibility. However, simple concatenation of audio and visual features is insufficient for effective fusion. Differences in feature representation, temporal resolution, and reliability mean fixed fusion strategies often fail to capture the dynamic relationship between modalities. Moreover, real-world visual inputs may suffer from blur, occlusion, pose variation, or detection errors, exacerbating modality imbalance.

Generative methods, such as diffusion-based models, can improve speech naturalness and detail restoration but require multi-step sampling, resulting in high inference latency and computational cost. FlowAVSE reduces this overhead via single-step conditional flow matching, achieving efficiency; however, it remains constrained by insufficient high-frequency recovery, limited waveform fidelity, and suboptimal utilization of visual cues under challenging noise.

To address these limitations, we propose FAV-DenoiseNet, a two-stage audio–visual speech enhancement framework that combines discriminative prior denoising with conditional residual flow refinement. The first stage suppresses dominant noise and generates a structurally stable speech prior. The second stage focuses on residual compensation between the Stage 1 output and the clean spectrum rather than predicting the entire spectrum directly. Residuals are estimated from zero-residual initialization using single-step inference, and multi-scale cross-attention enables audio features to adaptively leverage lip-motion cues across different resolutions. This two-stage design decouples coarse structure recovery from fine residual refinement, reducing the learning difficulty of generative enhancement and more effectively exploiting visual guidance compared to single-stage approaches such as FlowAVSE.

In experiments, FAV-DenoiseNet is trained primarily on GRID and evaluated on a VoxCeleb2 test subset for cross-dataset generalization, with no speaker overlap between training and test sets. Compared with state-of-the-art methods including FlowAVSE and AVDiffuSS, it consistently improves PESQ, ESTOI, and SI-SDR while maintaining single-step inference efficiency (RTF = 0.086), demonstrating a balanced improvement in speech quality, intelligibility, signal fidelity, and real-time processing.

The main contributions of this paper are summarized as follows:

1. This paper reformulates audio–visual speech enhancement as a prior-conditioned residual refinement problem. Instead of directly mapping noisy observations to the clean spectrum, the proposed framework first obtains a structurally stable speech prior and then refines the residual difference between this prior and the clean target.
2. A conditional residual flow-matching module is introduced for Stage-2 refinement. The flow-matching network estimates the residual correction from zero-residual initialization to the target residual, enabling single-step inference while reducing the learning difficulty of full-spectrum generative enhancement.
3. A multi-scale cross-modal attention mechanism is designed to inject lip-motion cues into the residual refinement process at different resolutions, providing adaptive visual guidance for speech-consistent local detail restoration under low-SNR conditions.

## 2. Related Work

### 2.1. Traditional and Single-Modal Speech Enhancement

Early speech enhancement studies were mainly based on statistical signal processing frameworks. Representative methods include the minimum mean-square error short-time spectral amplitude estimator (MMSE-STSA) [1] and spectral enhancement methods based on a priori signal-to-noise ratio (SNR) estimation [2]. These methods usually rely on relatively strong assumptions about noise statistics. Although they achieve reasonable performance in stationary noise environments, they often suffer from residual noise and speech distortion in complex non-stationary noise conditions, resulting in limited robustness.

With the development of deep learning, speech enhancement has gradually shifted toward data-driven supervised modeling. From the perspective of supervised speech separation, learning models, training objectives, and acoustic feature design have become key factors affecting enhancement performance [3]. The combination of deep neural networks with time–frequency masking targets, such as the ideal binary mask (IBM) and ideal ratio mask (IRM), has significantly improved single-channel speech enhancement. Weninger et al. explored long short-term memory (LSTM) networks for single-channel speech enhancement, in which time–frequency masks are predicted to suppress background noise, and the enhanced speech is used as a front-end for noise-robust automatic speech recognition (ASR). This study demonstrated the impact of speech enhancement on downstream recognition performance [4].

Subsequently, Tan et al. proposed the convolutional recurrent network (CRN), which adopts a convolutional encoder–decoder structure to extract local time–frequency features and incorporates an LSTM module to model temporal dependencies. This design enables effective real-time speech enhancement while maintaining causality [5]. Compared with methods relying solely on recurrent structures, CRN achieves a better balance between model size and enhancement performance. However, these methods still mainly depend on audio-only input. Under low-SNR or complex-noise conditions, they are prone to speech-detail loss and to insufficient recovery of high-frequency information.

### 2.2. Complex Spectral Modeling and Efficient Architectures

To further improve speech restoration capability, subsequent studies have explored several directions, including complex spectral modeling, long-range dependency modeling, and efficient architectural design. Based on CRN, Tan et al. further proposed the gated convolutional recurrent network (GCRN), extending the modeling objective from conventional magnitude-spectrum estimation to complex spectral mapping. Specifically, GCRN directly predicts the real and imaginary components of the speech spectrum, thereby recovering both magnitude and phase information [6]. Compared with methods that only process the magnitude spectrum, complex spectral modeling can better preserve the structural consistency of speech and, to some extent, alleviate the performance limitations caused by directly reusing the noisy phase.

To address long-term contextual modeling, various enhancement architectures combining convolutional and recurrent structures have been proposed. Gated residual convolutional network-based methods enhance temporal information modeling capability through gating mechanisms, dilated convolutions, and residual connections [7]. Strake et al. proposed the fully convolutional recurrent network (FCRN), which replaces the conventional fully connected LSTM with ConvLSTM. This design reduces the number of parameters while preserving the feature-map structure, enabling more effective modeling of speech harmonic structures [8]. Meanwhile, two-stage time-domain convolutional networks decompose the enhancement process into distinct stages and progressively improve enhancement accuracy [9]. Residual learning, introduced by He et al. [10], provides the architectural basis for the ResNet-based visual encoder adopted in this study.

On this basis, Zhao et al. proposed the Frequency Recurrent CRN (FRCRN) to overcome the limitations of conventional CRN/FCRN models in long-range modeling along the frequency dimension [11]. During the convolutional encoder–decoder process, FRCRN introduces recurrent modeling along the frequency axis and combines complex-domain cIRM prediction with joint loss optimization, achieving strong performance in wideband speech enhancement. This indicates that in addition to temporal modeling, explicit recurrent modeling along the frequency dimension is also beneficial for improving the capability to represent complex spectra.

At the same time, Transformer architectures have gradually been introduced into speech enhancement tasks. Self-attention-based methods can model long-range dependencies more effectively, but standard Transformers often suffer from a large number of parameters and insufficient modeling of local details [12]. To address this issue, Zhang Tianqi et al. proposed an efficient Transformer that uses time–frequency spatial attention and dual-branch attention fusion to achieve promising enhancement performance with a relatively small parameter size [13]. In addition, Wang Jingrun et al. proposed GFT-Conformer, which aims to reduce the computational cost of complex spectral modeling by using graph-frequency-domain representations while considering both local and global dependencies [14]. TF-GridNet further combines full-band, sub-band, and cross-frame self-attention mechanisms, demonstrating strong capability in complex time–frequency domain modeling [15].

Overall, complex spectral modeling and efficient architectural design have significantly promoted the development of single-modal speech enhancement. However, in extremely noisy environments, methods relying solely on audio input remain vulnerable to acoustic degradation.

### 2.3. Audio–Visual Speech Enhancement

Unlike audio signals, visual information is generally not directly affected by acoustic noise. The speaker’s lip movements, changes in mouth shape, and facial dynamics are closely related to speech articulation and can provide complementary constraints for speech enhancement. Michelsanti et al. provided a systematic overview of deep learning-based audio–visual speech enhancement and separation methods, pointing out that visual feature design, fusion strategies, and training targets are key factors affecting AVSE performance [16].

Early AVSE methods mostly relied on fixed fusion strategies. Hou et al. proposed AVDCNN, where independent audio and visual CNN streams are used to extract modality-specific features, which are then fused in a joint network. The model is optimized using multi-task learning to jointly address speech enhancement and visual reconstruction [17]. Experimental results show that incorporating visual information can effectively improve speech enhancement performance under highly noisy conditions. However, most early AVSE methods are developed under controlled scenarios and rely on fixed fusion frameworks, with limited consideration of modality imbalance in complex environments.

Beyond methods that directly use lip images, Morrone et al. proposed a speaker-independent AVSE method based on facial landmark motion [18]. This method uses an independently trained facial landmark detector to extract landmark motion features and employs an LSTM network to predict time–frequency masks. It achieves speaker-independent enhancement in multi-speaker scenarios on the GRID and TCD-TIMIT datasets, suggesting that explicit geometric motion features can still be beneficial when audio–visual training data are limited.

With the development of multimodal learning, the research focus has gradually shifted from simple feature concatenation to dynamic fusion mechanisms. Liu et al. proposed multiplicative combination and modality selection mechanisms to dynamically adjust the contribution of each modality according to the input sample, thereby alleviating the negative impact of weak modalities [19]. Building on this idea, Xu et al. proposed MHCA-AVCRN, which enhances the dynamic interaction between audio and visual modalities through multi-layer early fusion and multi-head cross-attention mechanisms [20]. Chuang et al. proposed LiteAVSE to improve online inference efficiency through visual compression and lightweight design [21]. Ren et al. further introduced a plug-in post-processing classifier (PPC) to mitigate target-speaker confusion caused by degraded visual quality and training-test mismatch, thereby improving model robustness [22].

Overall, existing AVSE methods have evolved from fixed fusion toward dynamic fusion and lightweight design. However, in real-world scenarios, they remain susceptible to fluctuations in visual quality, modality imbalance, and data distribution shifts.

### 2.4. Generative Audio–Visual Speech Enhancement

In addition to traditional discriminative methods, generative models have recently been introduced into audio–visual speech enhancement tasks. Compared with discriminative methods that directly predict masks or spectra, generative models can more effectively model the distribution of clean speech and therefore offer advantages in speech naturalness and detail restoration.

Lee et al. proposed AVDiffuSS, which introduces diffusion models into the audio–visual speech separation task and incorporates visual conditions into the diffusion process through a cross-attention mechanism, thereby enhancing audio–visual alignment [23]. Experimental results show that this method achieves favorable performance in terms of speech naturalness and intelligibility. This also indicates that diffusion-based generative models have the potential to alleviate over-suppression and perceptual distortion problems commonly observed in traditional enhancement methods. However, diffusion models typically rely on multi-step iterative sampling, leading to high inference complexity and practical limitations in real-time applications.

To reduce the inference cost of generative models, Jung et al. further proposed FlowAVSE, which introduces conditional flow matching into the audio–visual speech enhancement task [24]. While retaining the quality advantages of generative models, this method reduces the sampling process to a single inference step and reduces model complexity through a lightweight U-Net design. Experimental results show that FlowAVSE achieves a good balance between inference efficiency and enhancement performance.

Overall, generative AVSE is gradually evolving from diffusion-based paradigms, which often achieve high quality at high computational cost, toward flow-matching paradigms that better balance enhancement performance and inference efficiency. Nevertheless, existing methods still suffer from insufficient recovery of high-frequency details, limited fidelity to waveform structure, and insufficient exploitation of visual features in complex noise environments.

### 2.5. Cross-Modal Self-Supervised Representation Learning

In recent years, self-supervised learning has been widely applied to speech processing and multimodal learning tasks. HuBERT learns robust audio representations by combining clustering with masked prediction and demonstrates strong generalization in low-resource settings [25]. However, its modeling scope is limited to the audio modality.

Building on this idea, AV-HuBERT exploits the correspondence between audio signals and lip movements for cross-modal self-supervised pre-training and learns shared cross-modal representations through masked multimodal cluster prediction [26]. Experimental results show that AV-HuBERT significantly improves performance in lip reading and speech recognition. These results suggest that cross-modal self-supervised learning can provide stable shared priors and robust representations for audio–visual tasks.

Nevertheless, existing self-supervised methods are mainly evaluated on speech recognition and visual speech understanding tasks. Their stability and generalization in real-world AVSE scenarios with complex noise conditions remain insufficiently explored.

### 2.6. Evaluation Protocols and Metrics

Benchmark design and evaluation protocols are also important for comparing speech enhancement systems. The ICASSP 2022 Deep Noise Suppression Challenge provided a representative benchmark for evaluating noise suppression methods under diverse conditions [27]. For subjective evaluation, Naderi and Cutler investigated crowdsourced listening tests for noise suppression algorithms, supporting scalable perceptual assessment [28].

Objective evaluation commonly considers speech intelligibility, perceptual quality, and signal fidelity. ESTOI assesses intelligibility under modulated noise conditions [29], PESQ estimates perceptual speech quality [30], and SI-SDR evaluates scale-invariant signal distortion [31]. Together, these complementary metrics provide a more comprehensive evaluation of speech enhancement performance.

### 2.7. Discussion and Motivation

Overall, existing studies have gradually evolved from traditional statistical speech enhancement to supervised single-modal modeling, and further to complex spectral mapping, dynamic audio–visual fusion, and generative audio–visual enhancement. Although substantial progress has been made in speech enhancement, several challenges remain in complex real-world scenarios. Specifically, speech-detail recovery under extremely noisy conditions remains limited, fluctuations in visual quality may aggravate modality imbalance, and some generative models, despite their advantages in speech naturalness, introduce high inference costs, making them less suitable for real-time deployment [12,19,21,23,24].

To address these issues, this paper proposes FAV-DenoiseNet, a two-stage audio–visual speech enhancement model based on conditional flow matching and multi-scale cross-modal fusion. In Stage 1, a discriminative prior denoising module is employed to recover a relatively stable speech structure, thereby improving waveform fidelity in the presence of complex noise. In Stage 2, residual noise is further suppressed and speech details are restored through conditional flow matching, achieving a balance between speech naturalness and structural consistency. Meanwhile, a multi-scale cross-attention module is introduced into the generative stage, enabling audio features to adaptively capture articulation-related visual information and improving audio–visual collaborative modeling under low-SNR conditions. While maintaining strong enhancement performance, the proposed model also considers inference efficiency and practical deployment requirements through a lightweight design and single-step inference.

## 3. Methodology

### 3.1. Problem Formulation and Input Representation

This paper studied the task of audio–visual speech enhancement, which aimed to recover clean speech under the joint condition of noisy speech and the corresponding lip video sequence. Let y denote the noisy speech, x denote the clean speech, and V denote the temporally synchronized lip video sequence. The audio–visual enhancement model is denoted as $M_{AV}(\cdot )$, which maps the noisy speech and the visual sequence to an enhanced speech estimate:

(1) $$\hat{x}=M_{AV}\left(y,V\right).$$

where $\hat{x}$ denotes the enhanced speech estimate and $M_{AV}$ represents the audio–visual speech enhancement mapping.

For the audio input, the noisy speech was first resampled to 16 kHz and then transformed into the complex time–frequency domain using the short-time Fourier transform (STFT). The resulting complex spectral representation is given by the following:

(2) $$Y=STFT\left(y\right)\in C^{F\times T},$$

where F and T denote the frequency dimension and the number of time frames, respectively, using the complex spectrum as input preserves both magnitude and phase information of speech, thereby providing a more complete representation of the speech structure.

For the visual input, this paper adopted the lip image sequence temporally aligned with the audio signal as the visual modality information, denoted as follows:

(3) $$V=\{v_{1},v_{2},\ldots ,v_{L}\}.$$

Here, L denotes the number of lip frames in the visual sequence, and each lip image frame describes the mouth-motion state during speech, providing the model with auxiliary constraints on the articulation content.

### 3.2. Audio Front-End Representation

Based on the complex spectrum defined in Equation (2), this section specifies the audio front-end processing used before network input, including the STFT configuration and magnitude compression.

The STFT was performed with a Hann window. The FFT size was set to 512, the window length was 32 ms, and the frame shift was 10 ms, corresponding to a hop length of 160 samples. At a sampling rate of 16 kHz, this setting produced a one-sided STFT spectrum with 257 frequency bins for each time frame, i.e., a spectrum of size 257 × T before frequency-bin cropping. To satisfy the multi-scale downsampling and upsampling requirements of the subsequent U-Net architecture along the frequency dimension, the highest-frequency bin, i.e., the bin with index 257, was removed. Therefore, the model used the first 256 frequency bins as input, i.e., F = 256. During reconstruction, the complete one-sided spectrum was recovered via zero-padding before ISTFT.

To reduce the dominance of high-energy spectral components during training, magnitude compression was applied to the complex spectrum. Given the complex spectrum Y, the compressed representation is written as

(4) $$\tilde{Y}=\kappa \cdot |Y{|}^{e}exp(j∠Y),$$

where κ=0.15 and e=0.5. This transformation preserves the original phase and only applies nonlinear compression to the magnitude. It can improve the learnability of low-energy speech details to some extent and enhance the stability of model training. During the reconstruction stage, the corresponding inverse compression operation was applied, and the enhanced complex spectrum was converted back to the time-domain speech signal via the inverse short-time Fourier transform (ISTFT).

### 3.3. Visual Front-End Feature Extraction

In the visual branch, the visual front-end encoder consisted of a 3D convolutional front-end, a ResNet-based spatial encoder, a temporal convolutional network (TCN), and a one-dimensional convolutional projection module. Given the input lip sequence V = {v1,v2,…,vL}, the grayscale lip frames were first normalized by (x/255 − 0.4161)/0.1688 and processed by a 3D convolutional front-end. The 3D front-end used a 1→64 convolution with a kernel size of (5,7,7), stride of (1,2,2), and padding of (2,3,3), followed by batch normalization, ReLU activation, and max pooling with a kernel size of (1,3,3), stride of (1,2,2), and padding of (0,1,1). This module jointly modeled short-term temporal variation and local spatial structure in consecutive lip frames.

The resulting features were reshaped into frame-level feature maps and fed into the ResNet-based spatial encoder. The ResNet encoder consisted of residual convolutional layers with channel settings of 64→64, 64→64, 64→128, and 128→128, where the corresponding strides were 1, 2, 2, and 2. Each residual layer used 3 × 3 convolutional kernels and residual connections to extract frame-level visual representations. To further capture temporal lip-motion dynamics, a TCN with five depthwise-separable one-dimensional convolutional residual blocks was applied along the temporal axis. Each TCN block used a kernel size of 3, padding of 1, and 128 channels. Finally, a one-dimensional convolutional projection with kernel size 5 and padding 2 mapped the temporal features from 128 channels to a 64-dimensional visual representation fv. The obtained visual sequence remained temporally aligned with the audio features and was subsequently projected to the context dimension required by the cross-modal attention module.

Functionally, the visual front-end encoder followed a hierarchical representation learning strategy, covering local spatio-temporal feature extraction, frame-level spatial semantic modeling, and long-term temporal dynamic modeling. Specifically, the 3D convolution captured short-term local variations in lip movements, ResNet enhanced spatial structural representation, and the TCN further aggregated temporal context. This hierarchical design allowed visual articulatory priors to be effectively integrated into the enhancement network, improving the model’s ability to exploit complementary audio–visual cues.

### 3.4. Proposed Architecture

This paper proposed a two-stage progressive audio–visual speech enhancement framework. Given the complex spectrum of noisy speech and the temporally synchronized lip visual sequence, the model first performed coarse-grained denoising through a Stage-1 discriminative enhancement network. This stage aimed to recover the main speech structure and suppress dominant background noise, thereby providing a stable prior for the subsequent refinement stage. The Stage-2 conditional flow matching module then took the Stage-1 output as a conditional prior and performed fine-grained residual refinement, further improving speech detail restoration and perceptual quality.

**Figure 1:**
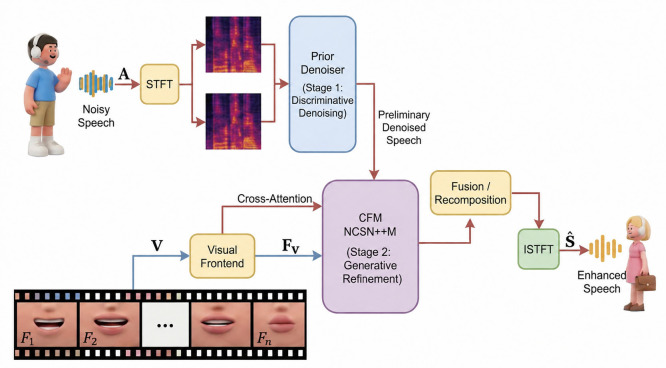
The overall architecture of FAV-DenoiseNet. The framework first obtains a preliminary enhanced speech signal using the Stage-1 prior denoiser, and then performs visually conditioned generative refinement in Stage 2, followed by spectral fusion and waveform reconstruction.

Compared with single-stage direct-mapping methods, the proposed framework decouples “structure recovery” from “detail refinement”. Stage 1 focuses on obtaining a stable preliminary enhancement result, while Stage 2 performs residual refinement for weak speech components, local spectral details, and continuity information. The visual branch does not directly predict high-frequency spectral values; instead, it provides articulation-related temporal cues, such as lip-motion patterns and mouth-shape changes. Through cross-modal attention, these visual cues constrain the residual refinement process and help the model distinguish speech-consistent local details from noise-dominated components under low-SNR conditions. As a result, the proposed model improves enhanced speech quality and intelligibility while maintaining high inference efficiency. The overall architecture of FAV-DenoiseNet is illustrated in Figure 1.

### 3.5. Stage 1: Prior Denoising and Coarse-Grained Enhancement

To reduce the modeling difficulty of the subsequent generative refinement stage, Stage 1 employed a pre-trained discriminative prior denoising module for preliminary enhancement of noisy speech. Rather than directly applying generative refinement to highly noisy and uncertain inputs, this stage performed coarse-grained restoration of the input speech and produced an intermediate enhanced result with a relatively stable structure and substantially reduced noise. The intermediate result was then used as conditional input for the Stage-2 refinement module. This staged design first restored the main speech structure, allowing for the subsequent generative module to focus on residual noise suppression, weak speech component recovery, and local detail compensation, thereby reducing the overall modeling complexity.

**Figure 2:**
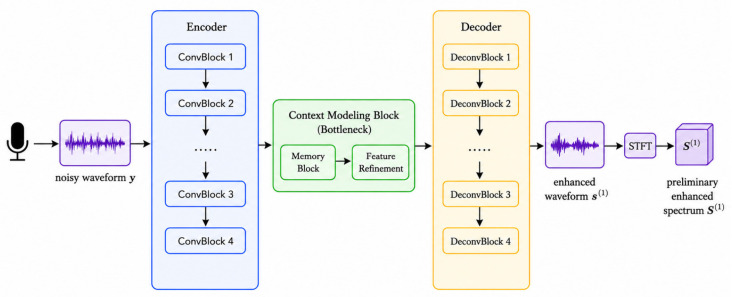
Stage-1 prior denoising and coarse-grained enhancement. The noisy waveform y is processed by the pre-trained discriminative encoder–decoder network, and the enhanced waveform $s^{\left(1\right)}$ is transformed by STFT to obtain an intermediate Stage-1 spectrum.

As shown in Figure 2, the noisy waveform y was directly fed into the Stage-1 prior denoising module. The encoder first extracted hierarchical speech features through stacked convolutional blocks. The context modeling block then refined the latent representation by capturing temporal dependencies, and the decoder reconstructed the enhanced waveform:

(5) $$s^{\left(1\right)}=P_{\theta }\left(y\right).$$

The enhanced waveform was then transformed into the frequency domain to obtain an intermediate Stage-1 complex spectrum:

(6) $$S_{STFT}^{\left(1\right)}=STFT(s^{\left(1\right)}).$$

In this way, the Stage-1 output was represented in the same complex spectral space as Stage 2, allowing for the conditional flow matching network to perform subsequent refinement within a unified representation domain.

The Stage-1 prior denoiser adopted a complex-domain encoder–decoder framework. The input signal was first transformed into a complex spectral representation through a time–frequency analysis module. The encoder then extracted multi-scale speech representations through a series of downsampling layers, enabling the network to capture complex noise patterns and time–frequency structures. To better model continuous speech dynamics, feed-forward sequential memory network (FSMN)-based contextual memory modules were inserted into the encoder, the intermediate bottleneck layer, and several decoder layers. These modules captured temporal dependencies between adjacent frames in multi-scale feature spaces. The decoder progressively restored high-resolution features through a symmetric upsampling path, while skip connections preserved shallow local details. In this way, the Stage-1 module produced a structurally stable coarse enhancement result.

In implementation, the Stage-1 prior denoiser adopted a waveform-based prior enhancement network with an internal complex-domain encoder–decoder structure. Its internal time–frequency analysis used a 640-point FFT with a window length of 640 and a hop size of 320. Complex FSMN modules with 128 hidden units were inserted into the encoder, bottleneck, and decoder stages, and SE layers with a reduction ratio of 8 were used for channel recalibration.

Let the encoder and decoder features at the l-th layer be denoted by $E_{l}$ and $D_{l}$, respectively. The feature restoration process at the corresponding layer can be formulated as follows:

(7) $$D_{l}=G_{l}\left(U\left(D_{l+1}\right),E_{l}\right),$$

where $U\left(\cdot \right)$ denotes the upsampling operation and $G_{l}\left(\cdot \right)$ represents the feature fusion and mapping function at the l-th layer. This multi-scale encoder–decoder structure jointly modeled the overall speech contour and preserved local details, making it suitable for coarse enhancement in Stage 1.

**Figure 3:**
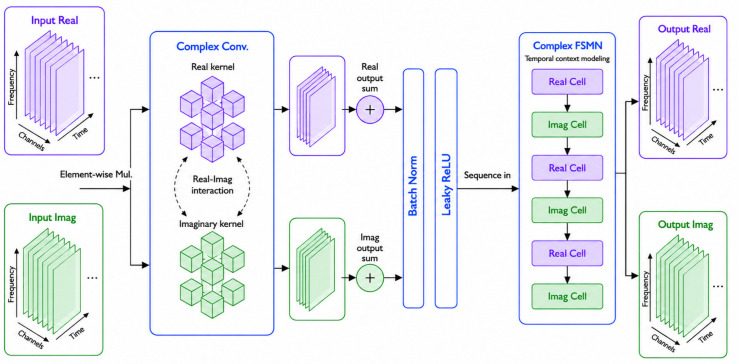
Complex convolutional FSMN block in Stage 1. It models complex spectral features and enhances temporal-context representation.

As shown in Figure 3, the real and imaginary feature maps were first processed by the complex convolution module, where real–imaginary interactions were modeled through separate real and imaginary kernels. The resulting real and imaginary outputs were then combined and passed through batch normalization and Leaky ReLU. After that, the complex FSMN module sequentially performed temporal context modeling and produced the updated real and imaginary feature maps. Specifically, each FSMN cell aggregated neighboring temporal features using learnable memory coefficients and incorporated the contextual response into the current frame representation.

Stage 1 did not rely solely on time-domain waveform mapping. Instead, its internal time–frequency analysis structure exploited both magnitude and phase information, providing a stable prior for subsequent complex spectral refinement. Let the noisy complex spectrum be denoted as

(8) $$Y=Y_{r}+jY_{i},$$

where $Y_{r}$ and $Y_{i}$ denote the real and imaginary components of the noisy spectrum, respectively. Accordingly, the enhanced spectrum produced by the Stage 1 can be expressed as

(9) $$S^{\left(1\right)}=S_{r}^{\left(1\right)}+jS_{i}^{\left(1\right)},$$

where $S_{r}^{\left(1\right)}$ and $S_{i}^{\left(1\right)}$ denote the real and imaginary components of the enhanced spectrum, respectively. Such time–frequency modeling helped preserve phase consistency and provided a reliable basis for complex spectral residual correction in Stage 2.

In the proposed two-stage framework, Stage 1 mainly served to provide a stable prior-enhanced result rather than directly generate the final enhanced speech. Therefore, during joint training, the parameters of the Stage-1 prior denoising module were kept frozen. This design avoided optimization instability caused by simultaneous updates of the two stages and allowed Stage 2 to learn a more targeted residual restoration mapping from stable conditional inputs. After Stage-1 processing, the main speech structure was generally recovered, and background noise was effectively suppressed. Nevertheless, high-frequency details, low-energy speech components, and local naturalness might still have been insufficiently restored. These aspects were therefore handled by the Stage-2 generative refinement module. Through this “coarse restoration followed by fine-grained enhancement” strategy, the model achieved a better balance between preserving the main speech structure and restoring natural speech details.

### 3.6. Representation-Domain Adaptation and Signal Flow

In the proposed two-stage cascaded framework, the Stage-1 discriminative prior denoising module and the Stage-2 generative refinement module operated on different signal representations. Therefore, representation-domain adaptation was required to bridge the two stages. Specifically, Stage 2 operated in the complex spectral domain and required the frequency dimension to be compatible with the multi-scale downsampling and upsampling structures of the network. For this reason, a one-sided complex spectrum with 256 frequency bins was used after removing the highest-frequency bin. In contrast, Stage 1 used the time-domain waveform as both its input and output representation. To connect these two domains, a representation-domain adaptation process was introduced to construct the Stage-2 noisy spectral input and the Stage-1 conditional spectral prior in a unified complex spectral representation.

**Figure 4:**
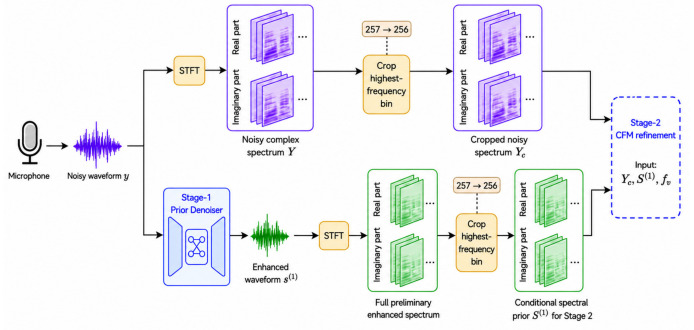
Representation-domain adaptation and signal flow between Stage 1 and Stage 2. The noisy waveform y is processed to obtain the cropped noisy spectrum $Y_{c}$ and the Stage-1 conditional spectral prior $S^{\left(1\right)}$ for Stage 2 refinement.

Let the noisy waveform input be denoted as y. To construct the spectral input for Stage 2, the waveform was first transformed into the complex spectral domain by STFT and then cropped along the frequency axis to obtain the noisy spectral input Yc. Meanwhile, the same waveform was fed into the Stage-1 prior denoising module, and the enhanced waveform was transformed into the spectral domain and cropped in the same manner to obtain the conditional spectral prior S(1) for Stage 2. In this way, both the noisy spectral input and the Stage-1 prior were aligned in the same complex spectral representation required by the Stage-2 refinement network. The detailed signal flow of this representation-domain adaptation process is illustrated in Figure 4.

As shown in Figure 4, the noisy waveform y was first transformed into the complex spectral domain and cropped along the frequency axis to obtain the Stage-2 noisy spectral input:

(10) $$Y_{c}=Crop(STFT(y))\in C^{F\times T}.$$

Meanwhile, the Stage-1 enhanced waveform $s^{\left(1\right)}$, obtained by the prior denoising module in Equation (5), was transformed into the frequency domain through STFT. The highest-frequency bin was then cropped to obtain a conditional prior that was consistent with the input format of the Stage-2 refinement network:

(11) $$S^{\left(1\right)}=Crop\left(STFT\left(s^{\left(1\right)}\right)\right)\in C^{F\times T}.$$

Here, Crop(⋅) denotes removing the highest-frequency bin from the one-sided STFT spectrum, yielding a complex spectrum with F=256 frequency bins.

In this way, the representation-domain adaptation process aligned both the noisy spectral input and the Stage-1 prior in the same complex spectral domain required by the Stage-2 refinement network. This design preserved the original processing mechanism of the Stage-1 module without modifying its internal structure while ensuring that the Stage-1 output was properly aligned with the complex spectral representation required by Stage 2. Consequently, a stable and reliable conditional prior was provided for subsequent generative refinement.

### 3.7. Stage 2: Generative Refinement and Audio–Visual Fusion

#### 3.7.1. Conditional Flow Matching

Although the Stage-1 discriminative denoising network can effectively suppress most background noise, fine-grained noise components may remain in its output, and the recovery of high-frequency details may be insufficient. To further improve speech perceptual quality and spectral detail restoration, this paper introduced a conditional residual flow-matching mechanism in Stage 2 to perform generative refinement of the Stage-1 output.

Unlike conventional diffusion models or standard flow-matching methods that directly generate clean speech from Gaussian noise, Stage 2 in this work did not predict the final clean speech. Instead, it took the preliminary enhanced complex spectrum $S^{\left(1\right)}$ produced by the Stage 1 as the condition and learned the residual representation between $S^{\left(1\right)}$ and the ground-truth clean speech spectrum X, which is defined as

(12) $$R=X-S^{\left(1\right)}.$$

This formulation had two advantages. On the one hand, since Stage 1 already recovered the main speech structure, Stage 2 did not need to generate the complete clean speech distribution from scratch but only needed to focus on the remaining errors. On the other hand, the residual target typically had lower energy and a more concentrated distribution, making it more suitable for rapid refinement with a lightweight flow-matching model.

During training, a time step t∼U(0,1) was sampled, and an interpolation state was constructed along the residual path from the zero residual to the target residual R. To avoid relying on a purely deterministic path, a small Gaussian perturbation with time-decaying variance was added during training, yielding

(13) $${\tilde{R}}_{t}=tR+\epsilon_{t},\;\epsilon_{t}∼N(0,\sigma_{min}^{2}(1-t{)}^{2}I).$$

In this residual formulation, the deterministic path was defined from the zero residual to the target residual R, with its deterministic component given by $R_{t}^{det}=tR$. Therefore, the corresponding target velocity was R. The perturbation term $\varepsilon_{t}$ in Equation (13) was used only during training to regularize intermediate residual states and vanished as t approached 1.

The Stage-2 network took the current residual state $R_{t}$, the time step t, the Stage-1 output $S^{\left(1\right)}$, and the visual conditional feature $f_{v}$ as inputs and predicted the residual flow vector field:

(14) $$v_{\theta }=F_{\theta }({\tilde{R}}_{t},t,S^{\left(1\right)},f_{v}).$$

The objective of $F_{\theta }$ was to approximate the correction direction toward the target residual. The corresponding flow matching loss is formulated as

(15) $$L_{FM}=E\left[{∥v_{\theta }-R∥}_{2}^{2}\right].$$

In addition to the main loss, several auxiliary losses were introduced to further constrain Stage 2’s refinement quality. First, the refined result is defined as

(16) $$X_{ref}=S^{\left(1\right)}+\hat{R},$$

where $\hat{R}$ denotes the complete residual estimate obtained from the predicted vector field. Based on this formulation, three auxiliary objectives were introduced to constrain the Stage-2 refinement process: (1) a residual reconstruction loss, which constrained the final refined result Xref to approach the ground-truth clean spectrum X; (2) an improvement-aware loss, which encouraged the Stage-2 output to reduce the reconstruction error relative to the Stage-1 prior; and (3) an auxiliary refinement loss, which imposed consistency constraints in both the log-magnitude spectral domain and the time domain.

Therefore, Stage 2 did not merely learn an abstract generative direction field. Instead, under the joint supervision of the main flow-matching loss and multiple reconstruction constraints, it focused on fine-grained compensation for residual errors left by Stage 1.

#### 3.7.2. Audio–Visual Generative Backbone Network

The Stage-2 refinement module adopted a generative backbone network modified from NCSN++. The overall structure followed a U-Net-style encoder–decoder framework, in which complex spectral features were modeled at multiple scales via layer-by-layer downsampling and upsampling. This design enabled the network to recover local time–frequency details and the global speech structure jointly. On this basis, the visual feature $f_{v}$ was provided to the Stage-2 generative network as a conditional input and injected into selected U-Net feature layers through cross-attention, enabling visual information to constrain audio refinement at multiple resolutions. Compared with directly concatenating audio and visual features, this design allowed the model to selectively exploit visual cues at different resolutions, thereby making better use of complementary information across modalities.

**Figure 5:**
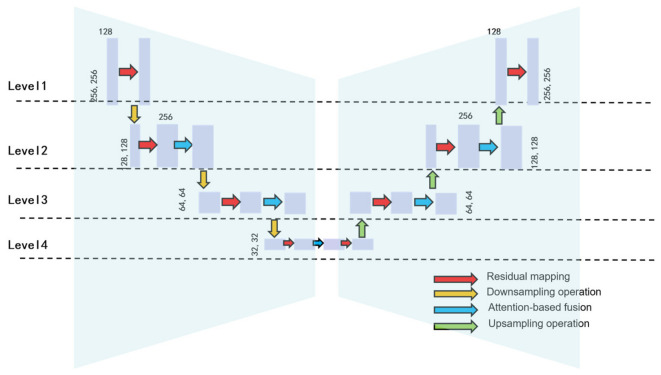
Multi-scale U-Net structure of the Stage-2 generative network. The colored arrows denote the signal-flow directions corresponding to ResNet processing, downsampling, attention, and upsampling operations.

The Stage-2 refinement network was implemented as a modified NCSN++ backbone with a base channel width of 128 and channel multipliers of (1, 2, 2, 2) across four resolution levels. The backbone followed an output-skip U-Net encoder–decoder architecture, with one residual block at each scale. Multi-scale cross-attention was applied at the 128, 64, and 32 resolution levels using 4 attention heads, a head dimension of 16, and a transformer depth of 1. The visual features were projected into a 256-dimensional context space and injected into the backbone for cross-modal fusion. The detailed multi-scale U-Net architecture of the Stage-2 generative network is illustrated in Figure 5.

The reason for adopting multi-scale cross-attention fusion was that audio features at different resolutions contain speech information at different levels. High-resolution features are more closely related to local details, short-term variations, and weak speech components. In contrast, low-resolution features are better suited to modeling long-range dependencies and the overall structure of speech. If visual conditions are introduced only at a single level, it is often difficult to capture both global constraints and local compensation simultaneously during decoding. In contrast, gradually injecting visual information at multiple scales allows visual cues to participate in global representation learning during the encoding stage and further constrain speech-related local detail restoration during the decoding stage. Therefore, this design was more suitable for audio–visual speech enhancement.

Let the audio feature at a certain scale be denoted as $H_{a}$, and the lip-motion feature extracted by the visual encoder be denoted as $f_{v}$. In the cross-attention module, the audio feature was projected into the query, while the visual feature was projected into the key and value:

(17) $$Q=W_{Q}H_{a},\;K=W_{K}f_{v},\;Z=W_{V}f_{v},$$

where $W_{Q}$, $W_{K}$, and $W_{V}$ are learnable linear projection matrices. The attention weights were then used to compute the conditional response of the visual features to the current audio representation:

(18) $$CrossAttn\left(H_{a},f_{v}\right)=Softmax\left(\frac{QK^{⊤}}{\sqrt{d}}\right)Z,$$

where d denotes the feature dimension. Through this process, the model can adaptively select articulation-related visual information based on the restoration requirements of the current audio features rather than indiscriminately feeding all visual features into subsequent convolutional layers. To evaluate the effectiveness of the cross-attention module, an ablation study was conducted, and the results are shown in Table 1.

In terms of the specific architecture, visual information was not fused at only a single position in the network. Instead, it was embedded into multiple resolution levels and interacted with multi-scale audio features. This design had two advantages. On the one hand, cross-modal attention in the encoding stage helped the model form a more stable global speech representation. On the other hand, cross-modal attention during the decoding stage imposed additional constraints on restoring local time–frequency details. Since the information provided by lip movements mainly served as auxiliary cues related to articulation content, rhythm variations, and semantic structure rather than precise spectral values, this multi-scale and stage-wise conditional injection strategy was usually more consistent with the practical requirements of audio–visual speech enhancement than one-shot fusion.

The role of the Stage-2 generative backbone was not only to perform generative refinement on the Stage-1 output but also to impose visual modality constraints during refinement continuously. In this way, the model can further correct residual noise, missing weak speech components, and local detail distortion in the preliminary enhanced result. With the multi-scale U-Net structure and the cross-attention fusion mechanism, the model can more effectively restore speech details and naturalness while preserving the main speech structure, thereby obtaining higher-quality enhanced speech.

#### 3.7.3. Cross-Modal Attention Fusion

To effectively fuse visual semantic information with audio features, this paper introduced an audio–visual cross-modal attention fusion module into the key resolution layers of the modified NCSN++. This module adopted a Transformer-based block as its basic unit and realized cross-modal information interaction through the sequential combination of self-attention, cross-attention, and a feed-forward network (FFN).

In this implementation, the audio feature at the current layer was first normalized using Group Normalization (GroupNorm). Then, average pooling was performed along the frequency dimension to compress the two-dimensional time–frequency feature into a one-dimensional temporal sequence representation. The resulting sequence was further mapped to the latent space corresponding to the current attention resolution through a linear projection. After this transformation, the audio feature was represented as a temporal token sequence suitable for attention computation.

The fusion module then sequentially performed the following three submodules:

Self-Attention: It modeled the dependencies within the audio temporal tokens and enhanced the ability of audio features to capture global context.

Cross-Attention: It used audio tokens as queries and visual features as keys and values, enabling conditional modulation of the audio representation by visual information.

Feed-Forward Network: It further applied a nonlinear transformation to the fused representation, improving the feature representation capability.

For each basic transformation block, let H denote the input audio token representation, and $H^{\left(1\right)}$, $H^{\left(2\right)}$, and $H^{\left(3\right)}$ denote the intermediate representations after self-attention, cross-attention, and the feed-forward network, respectively. The computation process can be summarized as

(19) $$H^{\left(1\right)}=H+SelfAttn\left(LN\left(H\right)\right),$$

(20) $$H^{\left(2\right)}=H^{\left(1\right)}+CrossAttn\left(LN\left(H^{\left(1\right)}\right),f_{v}\right),$$

(21) $$H^{\left(3\right)}=H^{\left(2\right)}+FFN\left(LN\left(H^{\left(2\right)}\right)\right).$$

where $LN\left(\cdot \right)$ denotes layer normalization. A residual connection was applied after each submodule to ensure stable gradient propagation and preserve the original audio features.

After cross-modal interaction, the fused temporal representation was projected back to the original feature-channel space via a linear projection, then injected into the current time–frequency feature map in a residual manner. Through this design, visual conditional information was not simply concatenated with audio features. Instead, it influenced the audio representation layer by layer through conditional modulation, allowing the speech refinement process to better conform to the corresponding lip-motion patterns while preserving the original acoustic structure.

Furthermore, the proposed fusion module was repeatedly introduced at several key resolution levels, namely 128, 64, and 32, forming a multi-scale audio–visual semantic injection mechanism. At coarser resolutions, the model primarily relied on visual cues to capture the overall trend in the speaker’s lip movements. At finer resolutions, visual information further constrained the restoration of local detail. This multi-scale cross-modal fusion strategy enabled Stage 2 to continuously receive visual conditions at different levels, thereby significantly improving the accuracy and naturalness of speech restoration under low-SNR conditions.

#### 3.7.4. One-Step-Inference-Dominated Residual-Controlled Fusion Strategy

Inference efficiency is an important factor in designing the refinement module in Stage 2. Traditional diffusion-based speech enhancement methods usually rely on multi-step sampling to gradually converge to the target distribution. Although they can achieve high-quality generation, they incur high inference costs. To continuously impose visual modality constraints during the refinement process, based on the recovery needs of the current audio features, rather than merely adding extra constraints at the decoding stage, this paper adopted a conditional flow matching framework to learn a time-conditional vector field from intermediate residual states to the target residuals. During inference, a one-step approximate update was used to obtain residual estimates, thereby reducing sampling complexity while maintaining refinement capability.

**Figure 6:**
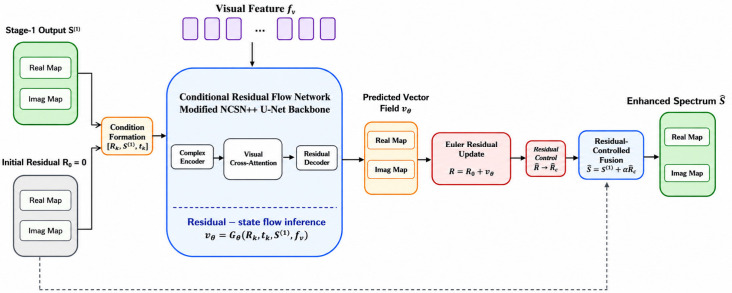
One-step residual update and residual-controlled fusion in Stage 2.

To better illustrate the one-step residual inference and residual-controlled fusion process, Figure 6 shows the Stage-2 refinement procedure. Given the coarse enhanced complex spectrum $S^{\left(1\right)}$ produced by Stage 1 and the visual conditional feature $f_{v}$, Stage 2 did not directly generate the complete clean complex spectrum. Instead, it estimated the residual between the Stage-1 output and the ground-truth clean speech. During inference, the zero residual was used as the initial state:

(22) $$R_{0}=0.$$

At the time step $t_{0}$, the residual flow network predicts the corresponding vector field:

(23) $$v_{\theta }=F_{\theta }\left(R_{0},t_{0},S^{\left(1\right)},f_{v}\right).$$

With one-step inference, the predicted residual is obtained by a single update from the zero residual state:

(24) $$\hat{R}=R_{0}+v_{\theta }.$$

This design preserved the ability of flow matching training to model continuous residual paths while avoiding multi-step iterative sampling during inference. As a result, Stage 2 could complete generative refinement with relatively low computational cost.

After obtaining the residual estimate, it was used as a corrective term to refine the Stage-1 output. To preserve the stable speech structure recovered by Stage 1, the correction injected into $S_{1}$ was bounded during fusion. Therefore, this paper adopted a residual-controlled fusion strategy, in which the compensation term produced by Stage 2 is constrained before being injected into the Stage-1 result. Specifically, a magnitude-based fusion bound was defined according to the average magnitude of the Stage-1 spectrum:

(25) $$C=\gamma \cdot Mean\left(\left|S^{\left(1\right)}\right|\right),$$

where C denotes the residual magnitude threshold and γ is the residual-control coefficient. In our implementation, γ = 0.6.

(26) $${\hat{R}}_{c}=\hat{R}\cdot min\left(1,\frac{C}{\left|\hat{R}\right|+\epsilon }\right),$$

where $\hat{R}$ denotes the residual estimated by the Stage-2 network, ${\hat{R}}_{c}$ denotes the bounded residual correction used for fusion, and ε = $10^{-6}$ is a small constant used to avoid numerical instability.

The residual-control coefficient was chosen to impose a moderate bound on the residual magnitude, preventing excessive correction while retaining sufficient capacity for Stage-2 refinement.

In addition to residual clipping, an adaptive fusion coefficient was introduced to control the contribution of the residual correction. The residual-to-prior magnitude ratio is defined as

(27) $$\rho =\frac{Mean\left(\left|{\hat{R}}_{c}\right|\right)}{Mean\left(\left|S^{\left(1\right)}\right|\right)+\epsilon }.$$

The adaptive fusion coefficient is then calculated as

(28) $$\alpha =clip\left(\alpha_{min}+\left(\alpha_{max}-\alpha_{min}\right)\rho ,\alpha_{min},\alpha_{max}\right),$$

where $\alpha_{min}$ and $\alpha_{max}$ denote the lower and upper bounds of the fusion coefficient, respectively. In our implementation, $\alpha_{min}=0.05$ and $\alpha_{max}=0.9$.

These bounds regulated the contribution of the residual correction during fusion, preventing the final estimate from being dominated by either the Stage-1 prior or the Stage-2 residual.

The final enhanced complex spectrum is expressed as

(29) $$\hat{S}=S^{\left(1\right)}+\alpha {\hat{R}}_{c}.$$

When α is close to $\alpha_{min}$, the model relies more on the stable Stage-1 prior. When α approaches $\alpha_{max}$, the Stage-2 residual correction contributes more strongly to the final enhanced spectrum. This operation controls the contribution of the residual correction during fusion, rather than changing the residual learning target of Stage 2. Therefore, the adaptive residual-controlled fusion strategy balances structural stability and residual detail compensation.

From a functional division perspective, Stage 1 provided a structurally stable basic complex spectrum, primarily ensuring the main speech contour and overall phase continuity. Stage 2 estimated the residual compensation term based on visual conditions, aiming to repair remaining detail loss and local distortion in the Stage-1 result. Residual-controlled fusion did not directly replace the Stage-1 output; instead, it applied a bounded generative correction on top of it, thereby striking a balance between stability and detail restoration. The fused complex spectrum $\hat{S}$ was finally reconstructed into the time-domain waveform through ISTFT and used as the final enhanced output of the model.

### 3.8. Optimization Objective and Training Strategy

A staged training strategy was adopted to optimize the two-stage enhancement model. The Stage-1 discriminative denoising module is pre-trained independently to learn the mapping from noisy speech to coarsely enhanced speech. During the Stage-2 training, the parameters of this module were kept frozen, and it participated only in forward computation as a stable prior enhancer. This strategy avoided optimization interference from simultaneous updates to the two stages and ensured that the generative refinement module was always built on relatively reliable conditional input.

In Stage 1, the training objective primarily focused on reconstruction error. Let the Stage-1 output be denoted as $s^{\left(1\right)}$, and the corresponding clean speech be denoted as x. The pre-training loss is defined as

(30) $$L_{stage1}={∥s^{\left(1\right)}-x∥}_{1}.$$

This loss was only used for the independent pre-training of Stage 1. During the training of Stage 2, the Stage-1 module no longer participated in parameter updates; therefore, $L_{stage1}$ was not included in the optimization objective of Stage 2. In other words, Stage 1 only provided a coarse-grained enhancement at this point rather than serving as an object to be optimized.

Stage 2 took the enhanced complex spectrum $s^{\left(1\right)}$ produced by Stage 1 and the visual feature fv as conditional inputs and learned generative refinement for the residual error defined in Equation (12).

During Stage-2 training, the residual flow network defined in Equation (14) was optimized using the flow-matching loss in Equation (15). For each sampled time step t, an intermediate residual state was constructed from the zero residual toward the target residual R, and the network predicted the corresponding correction direction. This loss directly constrained the network to learn the correction direction from the current residual state toward the target residual.

To prevent Stage 2 from only fitting the target at the vector-field level while ignoring the quality of the enhanced result itself, auxiliary reconstruction constraints were further introduced during training. Let the predicted residual of the Stage 2 be denoted as $\hat{R}$. The refined complex spectrum is defined as

(31) $$S^{\left(2\right)}=S^{\left(1\right)}+\hat{R}.$$

The residual reconstruction loss is defined as

(32) $$L_{rec}={∥S^{\left(2\right)}-X∥}_{1},$$

which constrains the refined complex spectrum to approach the ground-truth clean speech spectrum. The improvement-aware loss is defined as

(33) $$L_{imp}=max(0,||S^{\left(2\right)}-X|{|}_{1}-||S^{\left(1\right)}-X|{|}_{1}),$$

(34) $$L_{ref}=||log(1+|S^{\left(2\right)}|)-log(1+|X|)|{|}_{1}+||ISTFT(S^{\left(2\right)})-ISTFT(X)|{|}_{1},$$

which imposes consistency constraints in both the log-magnitude spectral domain and the time domain. The overall objective is formulated as

(35) $$L_{stage2}=L_{FM}+\lambda_{rec}L_{rec}+\lambda_{imp}L_{imp}+\lambda_{ref}L_{ref}$$

Here, $L_{rec}$, $L_{imp}$, and $L_{ref}$ denote the residual reconstruction loss, improvement-aware loss, and auxiliary refinement loss, respectively. The weights $\lambda_{rec}$, $\lambda_{imp}$, and $\lambda_{ref}$ are their corresponding contributions, while $L_{FM}$ remains the primary flow-matching objective.

The overall training procedure can be summarized into two stages. In the Stage 1, the discriminative denoiser was trained independently, and its parameters were updated using $L_{stage1}$. In Stage 2, the generative refinement and cross-modal fusion modules were trained, while Stage 1’s parameters were frozen. Stage 1 only provided $S^{\left(1\right)}$ as the conditional prior, and the optimization objectives were $L_{FM}$, $L_{rec}$, $L_{imp}$, and $L_{ref}$. This division enabled the model to exploit the stable, coarse-enhanced result from Stage 1 while allowing Stage 2 to perform targeted correction of residual noise, weak speech components, and local spectral details.

## 4. Experiments

In this section, the perceptual evaluation of speech quality (PESQ), extended short-time objective intelligibility (ESTOI), and scale-invariant signal-to-distortion ratio (SI-SDR) are selected to evaluate the effectiveness of FAV-DenoiseNet.

### 4.1. Datasets

FAV-DenoiseNet was trained on the GRID dataset, a widely recognized benchmark in audio–visual speech enhancement, which contained 30,786 speech segments. The dataset was split into training, validation, and test sets using an 8:1:1 ratio. To assess the model’s generalization and robustness, additional evaluation was conducted on a subset of VoxCeleb2, ensuring that the performance was tested across diverse speakers and recording conditions.

The VoxCeleb2 test set contained 476 segments. There was no speaker overlap between our training and test datasets.

### 4.2. Implementation Details

All clean and noisy speech signals were resampled to 16 kHz and amplitude-normalized to reduce volume differences among different recordings. The audio signals were processed using STFT, with a 512-point FFT, a 32 ms window length, a 10 ms frame shift (160 samples), and a Hann window to suppress spectral leakage. This produced a complex spectral representation with 257 one-sided frequency bins for each time frame, i.e., a spectrum of size 257 × T before cropping. To adapt the input format of U-Net-like networks, the 257th frequency bin was removed, and the first 256 frequency bins were retained as the model input. During training, fixed-length segments were randomly cropped along the temporal dimension to ensure batch alignment, while the frequency dimension was kept fixed at 256 bins. During inference, when the waveform was reconstructed through ISTFT, zero-padding was automatically applied to recover the 257th frequency bin, thereby ensuring the completeness of the reconstructed signal. The entire process was performed in the complex domain, which preserved complete magnitude and phase information.

In terms of training configuration, this paper adopted a two-stage training strategy. Stage 1 was a discriminative prior denoising module, while Stage 2 used an NCSN++ network with cross-modal attention as the conditional flow matching refinement network. During the Stage-2 training, the Stage-1 parameters were kept frozen, and only their enhanced complex spectrum was used as the conditional prior input. A dynamic signal-to-noise ratio (SNR) mixing strategy was adopted during training. Noise samples were drawn from the DEMAND database, covering domestic, office, public, transportation, and outdoor acoustic scenes. The used subsets included DKITCHEN, DLIVING, DWASHING, NFIELD, NPARK, NRIVER, OHALLWAY, OMEETING, OOFFICE, PCAFETER, PRESTO, PSTATION, SPSQUARE, STRAFFIC, TBUS, and TMETRO. For each clean utterance, the SNR was uniformly sampled from −5 dB to 15 dB. A noise segment was randomly selected and cropped to match the speech length; if it was shorter than the speech signal, it was repeated before cropping. The noise amplitude was then scaled according to the sampled SNR and added to the clean speech. The model was trained on a single Tesla V100S PCIe 32 GB GPU (NVIDIA Corporation, Santa Clara, CA, USA), with a batch size of 4 and 4 data-loading workers. The learning rate was set to $3\times 10^{-5}$, and the minimum time step $t_{\epsilon }$ was set to 0.03. The flow-matching loss used mean squared error, and the Stage-1 reconstruction loss was formulated as a combination of complex and magnitude spectral errors. In Stage 2, λ_rec, λ_imp, and λ_ref were set to 1.0, 0.2, and 0.1, respectively. During training, 10 validation samples were selected in each epoch for online evaluation.

Video features were extracted using a visual encoder to model the representation of lip-region frame sequences. For the visual input, the facial region was first detected from the original video, and a facial landmark detector was employed to localize the mouth area. Specifically, landmarks 48–67 were used to compute the mouth center and bounding box, and the lip region of interest (ROI) was cropped based on the mouth center. The size of the cropped region was determined by the maximum of the mouth width and height, with an extension factor of approximately 2.0 to fully cover the lip motion area. The cropped lip images were converted to grayscale and resized to 88 × 88 pixels. Video frame rates were standardized to 25 fps to ensure temporal alignment with the corresponding audio frames. In cases where landmark detection fails, the lower-center 88×88 region of the image was used as a fallback crop to maintain continuity of visual input. The processed visual input was then fed into the visual front-end encoder described in Section 3.3 to extract temporally aligned visual features, which were further projected to match the conditional feature dimension of the Stage-2 cross-attention module. The resulting visual feature sequence maintained temporal correspondence with the audio features and served as conditional input to the subsequent cross-attention mechanism, guiding the model to leverage lip motion information for speech enhancement.

To improve reproducibility, the main layer configurations and hyperparameter settings of FAV-DenoiseNet are summarized in Table 2.

### 4.3. Experimental Results

#### Overall Results

**Overall performance.** As shown in Table 3, the proposed method achieves strong overall performance on both the VoxCeleb2 and GRID test sets. On VoxCeleb2, FAV-DenoiseNet obtains a PESQ of 2.805, an ESTOI of 0.775, and an SI-SDR of 12.480 dB. On GRID, it achieves a PESQ of 3.157, an ESTOI of 0.876, and an SI-SDR of 13.281 dB. Compared with the representative generative audio–visual baselines, the proposed method achieves better overall enhancement performance while maintaining single-step inference efficiency.

**Ablation study on the FSMN module.** To further analyze the contribution of the Stage-1 prior denoising network, we conducted an ablation experiment on the FSMN module. The variant denoted as w/o FSMN removes the FSMN-based temporal context modeling component, while Stage1_only represents the complete Stage-1 denoising network. This experiment focuses on the Stage-1 module and aims to verify whether temporal context modeling can provide a more stable coarse-enhanced prior for the subsequent conditional flow matching refinement stage.

As shown in Table 4, removing the FSMN module leads to a substantial performance degradation on the VoxCeleb2 test set. Compared with Stage1_only, the w/o FSMN variant decreases from 2.658 to 1.558 in PESQ, from 0.776 to 0.520 in ESTOI, and from 11.874 dB to 2.238 dB in SI-SDR. These results indicate that FSMN-based temporal context modeling is important for preserving speech continuity and modeling temporal dependencies in the Stage-1 enhancement process. Therefore, the FSMN module helps generate a more reliable discriminative prior for the Stage-2 generative refinement network.

**Robustness under different SNR conditions.** To evaluate the robustness of FAV-DenoiseNet, we reported its performance on VoxCeleb2 and GRID under fixed SNR settings of −5 dB, 0 dB, 5 dB, 10 dB, and 15 dB, together with a mixed-SNR condition. The mixed condition follows the training setting, where the SNR is uniformly sampled from −5 dB to 15 dB. As shown in Table 5, FAV-DenoiseNet maintains reliable enhancement performance across different noise levels, with generally higher scores observed as the input SNR increases.

Under the challenging −5 dB condition, FAV-DenoiseNet still achieves PESQ scores of 2.519 and 2.669 on VoxCeleb2 and GRID, respectively. At 15 dB, it obtains the best fixed-SNR results, with PESQ, ESTOI, and SI-SDR scores of 3.562, 0.892, and 17.141 dB on VoxCeleb2 and 3.842, 0.962, and 16.425 dB on GRID, respectively. The mixed condition reflects the average behavior under the training SNR distribution.

**Comparison with representative audio–visual baselines.** To provide a controlled comparison under the same evaluation protocol, FAV-DenoiseNet is compared with two representative generative audio–visual baselines, AVDiffuSS and FlowAVSE. The comparison is limited to methods evaluated under the same preprocessing pipeline, inference setting, and objective metrics. Since AVDiffuSS is a representative generative audio–visual speech separation method and shares a similar audio–visual generative modeling paradigm with AVSE, it is included as a comparative baseline under the same evaluation protocol. As shown in Table 3, the proposed method achieves the best overall performance under the single-step inference setting. On VoxCeleb2, compared with FlowAVSE, FAV-DenoiseNet improves PESQ, ESTOI, and SI-SDR by 1.082, 0.069, and 2.442 dB, respectively. On GRID, the corresponding improvements are 1.156, 0.084, and 2.598 dB. Compared with AVDiffuSS, which requires 30 sampling steps, the proposed method also achieves higher objective scores.

**Figure 7:**
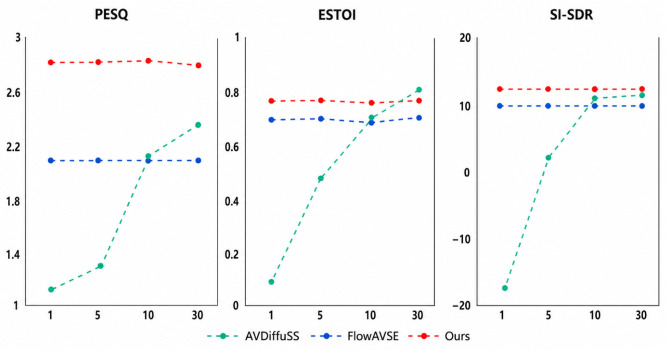
A comparison of PESQ, ESTOI, and SI-SDR on the VoxCeleb2 test set across different inference steps, including 1, 5, 10, and 30 steps. The proposed FAV-DenoiseNet achieves strong single-step performance and remains stable as the number of inference steps increases.

These results indicate that the proposed discriminative prior denoising and conditional flow matching refinement strategy can improve enhancement performance while maintaining generation quality and single-step inference efficiency. As shown in Table 3, the proposed method requires only one inference step and achieves an RTF of 0.086, which is nearly comparable to that of FlowAVSE with an RTF of 0.085 and is significantly better than the 30-step AVDiffuSS with an RTF of 1.532. This result indicates that FAV-DenoiseNet does not introduce significant inference overhead after incorporating the two-stage architecture and the multi-scale cross-modal attention mechanism while still maintaining high inference efficiency. Compared with diffusion-based methods that rely on multi-step sampling, the proposed method is better suited for real-time or near-real-time audio–visual speech enhancement scenarios. To further evaluate the effect of the number of inference steps, Figure 7 compares PESQ, ESTOI, and SI-SDR on the VoxCeleb2 test set using 1, 5, 10, and 30 inference steps.

**Ablation analysis.** Table 6 presents the experimental results of different model variants. Here, Stage1_only denotes using only the Stage-1 discriminative prior denoising module, Stage2_only denotes using only the Stage-2 generative refinement module, Audio-only denotes removing the visual input and retaining only the audio branch, and Ours denotes the full model. Bold values indicate the best performance for each metric.

**Subjective listening test.** To further evaluate perceptual speech quality, an online subjective listening test was conducted following the ITU-T P.835 protocol. Fifteen listeners participated, and 13 valid responses were retained after excluding incomplete or invalid submissions. Ten utterances from the VoxCeleb2 mixed-SNR test subset were selected, and five versions of each utterance were evaluated: Noisy, AVDiffuSS, FlowAVSE, FAV-DenoiseNet, and Clean, resulting in 50 audio samples. The method labels and file names were hidden, and all samples were randomly presented to each listener. Each sample was rated in terms of speech signal quality (SIG-MOS), background noise quality (BAK-MOS), and overall quality (OVRL-MOS) on a 1–5 scale. The final scores are reported as the mean and standard deviation over all valid ratings. The subjective listening test results are summarized in Table 7.

**Figure 8:**
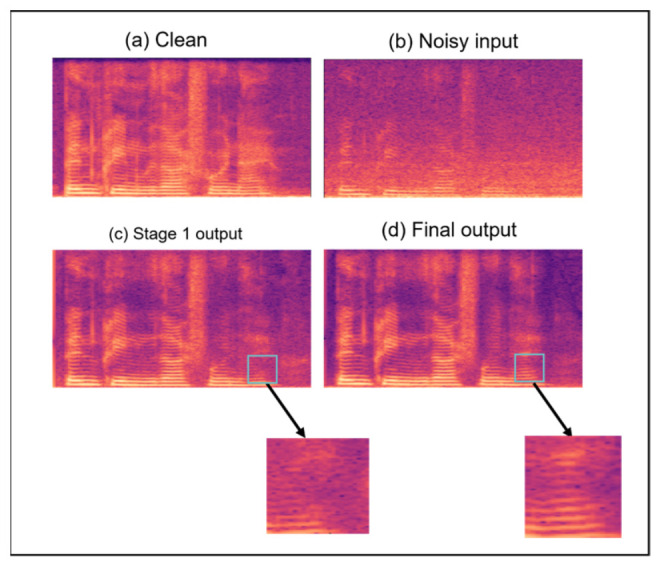
The spectrogram comparison of clean speech, noisy input, Stage-1 output, and final output. The enlarged regions show that Stage 1 preserves the main speech structure but still contains local residual artifacts, while the final output provides clearer spectral details after Stage-2 refinement.

**Spectrogram analysis.** Figure 8 presents a spectrogram comparison among clean speech, noisy input, Stage-1 output, and the final enhanced result on the VoxCeleb2 test set. As shown in the enlarged regions, the Stage-1 output recovers the main speech structure but still contains local residual artifacts and incomplete spectral details. After Stage-2 refinement, these regions become clearer and closer to the clean speech spectrogram. This result visually supports the necessity of the Stage-2 residual refinement.

**Figure 9:**
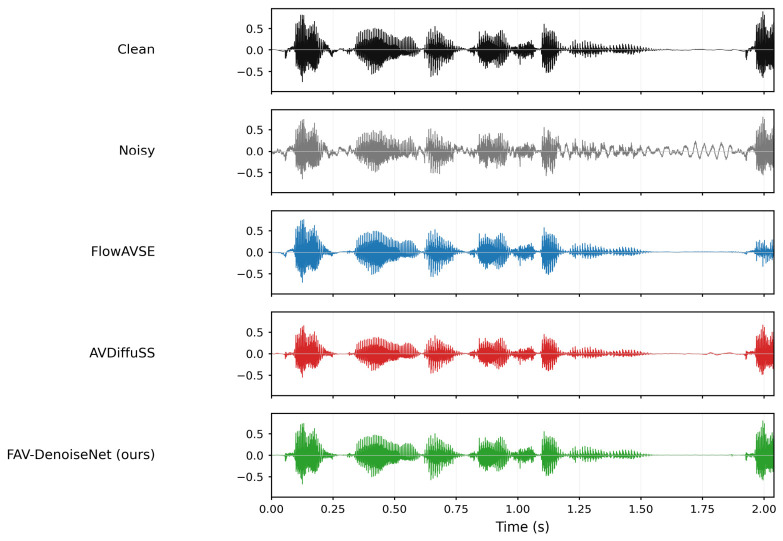
A waveform comparison of the clean reference, noisy input, FlowAVSE, AVDiffuSS, and FAV-DenoiseNet (ours) on the same representative test utterance.

**Waveform analysis.** Figure 9 compares the time-domain waveforms of the clean reference, noisy mixed input, and enhanced outputs produced by FlowAVSE, AVDiffuSS, and FAV-DenoiseNet under the same test utterance. Compared with the noisy input, the enhanced outputs suppress background-induced fluctuations to different degrees. In this representative utterance, FAV-DenoiseNet better preserves the speech envelope and local waveform structure, especially in the weak-speech and terminal speech regions, producing a waveform pattern closer to the clean reference.

## 5. Conclusions

To address insufficient detail recovery, inadequate utilization of visual information, and the high inference cost of generative models in existing audio–visual speech enhancement methods for complex noisy environments, this paper proposes FAV-DenoiseNet, a two-stage audio–visual speech enhancement model based on conditional flow matching and visual encoding. The proposed model first employs a discriminative prior denoising module to perform coarse-grained enhancement of noisy speech, producing a preliminary enhanced result with stable structure and controlled residual noise. Subsequently, a conditional flow matching mechanism is introduced in Stage 2, where the Stage-1 output is used as a conditional prior to further suppress residual noise and refine speech-related local spectral details and weak speech components.

Meanwhile, a multi-scale cross-attention fusion mechanism is incorporated into the generative refinement network, enabling the model to adaptively exploit lip-motion information at different resolution levels and thereby improve speech restoration under low-SNR conditions. By adopting the two-stage structure of “Stage-1 coarse enhancement + Stage-2 conditional flow matching refinement” and using visual features as conditional information in the generative refinement process, the proposed method effectively improves enhancement quality, speech intelligibility, and inference efficiency.

Experimental results show that FAV-DenoiseNet achieves favorable enhancement performance on both the VoxCeleb2 and GRID test sets. Specifically, on VoxCeleb2, the proposed method obtains a PESQ of 2.805, an ESTOI of 0.775, and an SI-SDR of 12.480 dB; on GRID, it achieves a PESQ of 3.157, an ESTOI of 0.876, and an SI-SDR of 13.281 dB. These results demonstrate that the proposed method achieves superior overall performance compared with baseline methods such as FlowAVSE. Ablation experiments further show that the Stage-1 discriminative prior is important for stabilizing the Stage-2 generative refinement process, while the visual modality provides effective articulatory priors for speech enhancement. Notably, the proposed method requires only one inference step and achieves an RTF of 0.086, maintaining strong enhancement performance while improving inference efficiency. Together, these findings suggest that FAV-DenoiseNet has promising potential for real-time or near-real-time audio–visual speech enhancement.

Overall, FAV-DenoiseNet achieves a favorable balance among enhancement quality, speech intelligibility, and inference efficiency through the joint design of discriminative prior denoising, conditional flow matching-based residual refinement, and multi-scale audio–visual fusion. Future work will focus on the following three aspects. First, more complex real-world noisy scenarios and broader cross-dataset evaluations should be introduced to further verify the robustness and generalization ability of the model in practical applications. Second, model compression of both the visual front-end and the generative refinement module should be explored to reduce model parameters and computational cost. Third, more extensive subjective listening tests and downstream automatic speech recognition evaluations can be incorporated to establish a more comprehensive evaluation framework for audio–visual speech enhancement.

## Figures and Tables

**Table 1:** The ablation results of the cross-attention module. Removing the cross-attention module leads to performance degradation, demonstrating its effectiveness in audio–visual fusion.

Method	VoxCeleb2		
	PESQ	ESTOI	SI-SDR
No-CrossAtt	2.556	0.760	10.809
with CrossAtt	2.805	0.775	12.480

**Table 2:** Main layer and hyperparameter settings of FAV-DenoiseNet.

Module	Main Layers/Structure	Key Settings
Audio front-end	STFT and complex spectral representation	16 kHz; 512-point FFT; 32 ms Hann window; 10 ms hop; 257 one-sided frequency bins cropped to 256 bins
Visual encoder	3D Conv + ResNet + TCN + 1D Conv projection	Lip ROI resized to 88 × 88; Conv3D 1 → 64 with kernel (5,7,7); ResNet residual blocks; 5 depthwise-separable TCN blocks; 64-dimensional visual representation projected to a 256-dimensional attention context
Stage-1 prior denoiser	Pre-trained complex-domain encoder–decoder with FSMN and SE modules	Internal FFT/window/hop: 640/640/320; FSMN hidden size 128; SE reduction ratio 8; frozen during Stage-2 training
Stage-2 CFM backbone	Modified NCSN++ U-Net	Base channels 128; channel multipliers (1,2,2,2); four resolutions 256/128/64/32; one residual block per scale
Cross-modal attention	Self-attention + cross-attention + FFN	Inserted at resolutions 128, 64, and 32; 4 heads; head dimension 16; transformer depth 1
Residual flow matching	Conditional residual vector-field prediction	Residual path from zero residual to target residual; t ~ U(0,1); t_eps = 0.03; sigma_min = 1 × 10^−4^; one-step inference
Residual-controlled fusion	Bounded residual correction	Residual-control coefficient γ = 0.6; adaptive fusion coefficient bounded by α_min = 0.05 and α_max = 0.9
Training	Two-stage optimization	Stage 1 pre-trained independently and frozen for Stage 2; Adam optimizer; learning rate 1 × 10^−4^; batch size 4; 40 epochs; gradient clipping 1.0
Loss weights	Stage-2 training objective	Flow-matching loss with MSE; λ_rec = 1.0, λ_imp = 0.2, λ_ref = 0.1

**Table 3:** Performance comparison with representative generative audio–visual baseline methods on the VoxCeleb2 and GRID datasets.

Method	RTF	Steps	VoxCeleb2			GRID		
			PESQ	ESTOI	SI-SDR	PESQ	ESTOI	SI-SDR
AVDiffuSS	1.532	30	2.023	0.760	11.508	1.979	0.722	9.622
AVDiffuSS	0.083	1	1.153	0.084	−15.272	1.452	0.263	3.481
FlowAVSE	0.085	1	1.723	0.706	10.038	2.001	0.792	10.683
Ours	0.086	1	2.805	0.775	12.480	3.157	0.876	13.281

**Note:** All compared methods are run on the same local device using the same preprocessing pipeline and test protocol. RTF is measured on the same local device and includes STFT/ISTFT and visual feature extraction.

**Table 4:** The ablation study of the FSMN module in the Stage-1 prior denoising network on the VoxCeleb2 test set.

Method	VoxCeleb2		
	PESQ	ESTOI	SI-SDR
w/o FSMN	1.558	0.520	2.238
Stage1_only	2.658	0.776	11.874

**Table 5:** The performance of FAV-DenoiseNet under different SNR conditions on the VoxCeleb2 and GRID datasets.

Method	SNR	VoxCeleb2			GRID		
		PESQ	ESTOI	SI-SDR	PESQ	ESTOI	SI-SDR
FAV-DenoiseNet	−5 dB	2.519	0.665	9.293	2.669	0.773	9.865
	0 dB	3.071	0.808	13.087	3.150	0.877	12.063
	Mixed	2.805	0.775	12.480	3.157	0.876	13.281
	5 dB	3.247	0.840	15.009	3.473	0.916	13.438
	10 dB	3.495	0.888	16.150	3.755	0.947	16.251
	15 dB	3.562	0.892	17.141	3.842	0.962	16.425

**Table 6:** The ablation results of different FAV-DenoiseNet variants on the VoxCeleb2 and GRID datasets.

Method	VoxCeleb2			GRID		
	PESQ	ESTOI	SI-SDR	PESQ	ESTOI	SI-SDR
Stage1_only	2.658	0.776	11.874	3.031	0.851	11.768
Stage2_only	1.635	0.565	0.488	1.884	0.681	0.868
Audio-only	2.678	0.756	11.536	2.902	0.792	11.885
Ours	2.805	0.775	12.480	3.157	0.876	13.281

**Table 7:** Subjective listening test results on VoxCeleb2 under the mixed-SNR condition following the ITU-T P.835 protocol.

Method	SIG-MOS	SIG-std	BAK-MOS	BAK-std	OVRL-MOS	OVRL-std
Clean	4.800	0.545	4.733	0.445	4.733	0.528
Noisy	4.356	0.948	2.986	0.577	3.658	0.472
AVDiffuSS	4.411	0.562	3.539	0.474	3.892	0.498
FlowAVSE	4.562	0.529	3.973	0.490	4.027	0.520
FAV-DenoiseNet	4.732	0.531	4.249	0.503	4.475	0.637

## Data Availability

The original contributions presented in this study are included in the article. Further inquiries can be directed to the corresponding author.
